# Tetraspanin CD82 Regulates the Spatiotemporal Dynamics of PKCα in Acute Myeloid Leukemia

**DOI:** 10.1038/srep29859

**Published:** 2016-07-15

**Authors:** Christina M. Termini, Keith A. Lidke, Jennifer M. Gillette

**Affiliations:** 1 Department of Pathology, University of New Mexico Health Sciences Center, University of New Mexico, MSC 08-4640, Albuquerque, NM 87131, USA; 2 Department of Physics and Astronomy, University of New Mexico, MSC 07-4220, Albuquerque, NM 87131, USA.

## Abstract

Patients with acute myeloid leukemia (AML) have increased myeloid cells within their bone marrow that exhibit aberrant signaling. Therefore, therapeutic targets that modulate disrupted signaling cascades are of significant interest. In this study, we demonstrate that the tetraspanin membrane scaffold, CD82, regulates protein kinase c alpha (PKCα)-mediated signaling critical for AML progression. Utilizing a palmitoylation mutant form of CD82 with disrupted membrane organization, we find that the CD82 scaffold controls PKCα expression and activation. Combining single molecule and ensemble imaging measurements, we determine that CD82 stabilizes PKCα activation at the membrane and regulates the size of PKCα membrane clusters. Further evaluation of downstream effector signaling identified robust and sustained activation of ERK1/2 upon CD82 overexpression that results in enhanced AML colony formation. Together, these data propose a mechanism where CD82 membrane organization regulates sustained PKCα signaling that results in an aggressive leukemia phenotype. These observations suggest that the CD82 scaffold may be a potential therapeutic target for attenuating aberrant signal transduction in AML.

Acute myeloid leukemia (AML), the most common acute leukemia affecting adults, is characterized by increased immature myeloid blasts within the bone marrow, which interferes with normal hematopoiesis^1^. While an increasing number of chemotherapy drugs are being made available, AML remains a highly fatal disease due to its significant relapse rate following standard treatment^2^. Modeling studies have demonstrated that the expression and activation of signaling molecules can be used to predict AML patient remission attainment, relapse, and survival^3^. For example, increased expression of the protein kinase C (PKC) isoform PKCα correlates with poor survival in AML patients^4^. Therefore, therapeutic targeting of specific aberrant signaling in AML may be used to treat this aggressive disease.

The PKC family of enzymes are serine/threonine kinases that can be further classified into conventional, novel, and atypical PKCs^5^. The conventional PKC isoforms include PKCα, β1, β2 and γ, all of which require Ca^2+^ and diacylglycerol (DAG) to become activated. Upon activation, PKC is initially phosphorylated within the cytoplasm and translocates to the plasma membrane following full phosphorylation. This translocation process is controlled by DAG production but may be bypassed with the use of the PKC activator, phorbol 12-myristate 13-acetate (PMA)^6^. PKC activation initiates various signaling responses such as the activation of Rac1, RhoA, and the mitogen activated protein kinases (MAPK) signaling cascades^6^,^7^,^8^,^9^. As such, PKC activation controls many basic cellular processes including adhesion, migration, and proliferation, which all contribute to cancer progression.

In AML patients, PKCα gene expression is upregulated when compared to CD34^+^ normal donors^10^. Furthermore, treating AML cell lines with the PKC inhibitor, enzastaurin, blocks the phosphorylation of PKCα and its downstream target, ERK, and also prevents PKCα membrane recruitment^10^. Additional work suggests that increased levels of phospho-PKC are correlated with increased AML cell viability^11^. However, the molecules and mechanisms that control PKC activation and downstream signaling remain poorly defined.

Tetraspanins serve as molecular scaffolds within the plasma membrane to generate highly organized membrane domains, termed tetraspanin enriched microdomains (TEMs)^12^,^13^. TEMs consist of interactions between tetraspanins and with other membrane proteins including integrins and signaling receptors such as the epidermal growth factor receptor (EGFR) and c-kit^14^,^15^,^16^. The maintenance of TEMs promote cellular functions including cell adhesion, migration, and proliferation^17^,^18^,^19^. The palmitoylation of tetraspanins regulate TEM organization through the control of protein-protein interactions^14^,^20^,^21^, which can in turn mediate cellular signaling. For example, expression of the palmitoylation deficient form of CD151 weakens tetraspanin association with integrins, resulting in diminished AKT phosphorylation in response to laminin-5 engagement^14^. Moreover, inhibition of CD81 palmitoylation reduced signaling in B cells, as assessed by PLCγ2 and VAV phosphorylation^22^. Therefore, tetraspanin palmitoylation can control various aspects of cellular signaling.

In addition to membrane proteins, tetraspanins interact with cytosolic proteins such as the serine/threonine binding protein 14-3-3^23^ and G protein subunits^24^. Moreover, previous work established that CD151 assists in the recruitment of Rac1 to the plasma membrane, in addition to associating with PKCα^23^,^24^,^25^. Interestingly, tetraspanins CD9, CD81 and CD82 were shown to associate with PKCα upon PMA activation^26^, and coimmunoprecipitation studies with CD9 and CD151 detected PKCα associations. In the present study, we focus on identifying how this tetraspanin association modulates PKC signaling, with a specific emphasis on CD82.

Although it has been demonstrated that many tetraspanins can interact with PKCα, we have chosen to focus on CD82 due to previous work demonstrating that CD82 is upregulated in several human leukemias, including AML^27^. Recently, CD82 upregulation was identified in chemotherapy-resistant CD34^+^/CD38^−^ AML cells^28^, which are often the cells responsible for disease relapse. The objective of this study is to determine how the CD82 scaffold and its membrane organization regulate PKCα-mediated signaling and influence AML progression. Using a combination of single molecule and ensemble imaging techniques, we find that CD82 modulates the spatial and temporal dynamics of PKCα signaling in AML cells. Our data demonstrate that the molecular organization of CD82 regulates PKCα stabilization and clustering at the plasma membrane, which controls downstream ERK signaling and AML colony formation. Together, our findings suggest that CD82 organization may be a suitable target for controlling AML progression through its regulation of PKCα signaling.

## Results

### The CD82 scaffold regulates PKCα expression and activation

**Figure 1:**
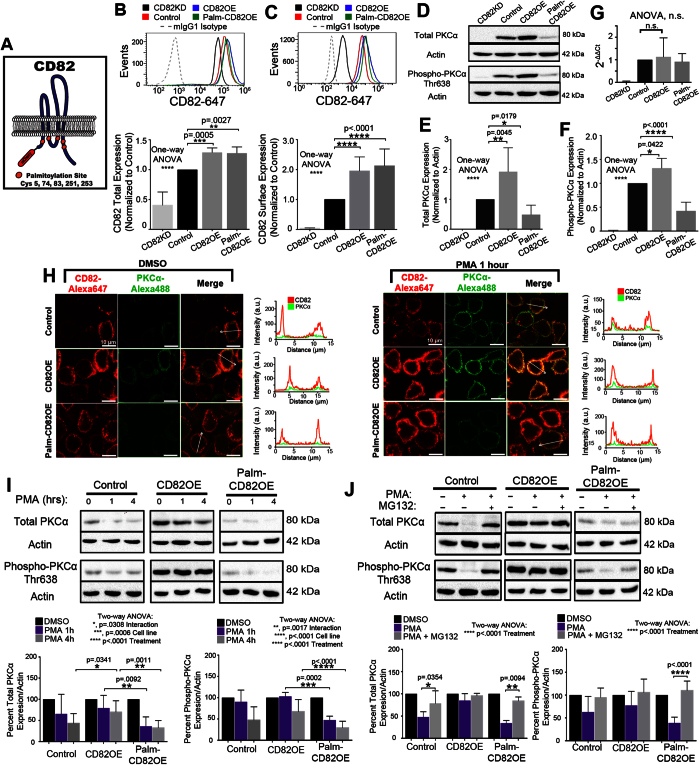
The CD82 scaffold regulates PKCα expression and activation. (**A**) Cartoon depicting mutated palmitoylation sites within CD82 and mCherry fusion. Flow cytometry analysis of (**B**) total and (**C**) surface CD82 expression using CD82KD, control, CD82OE, and Palm-CD82OE KG1a cells (Biolegend, ASL-24). (n ≥ 3 experiments; error bars indicate SD; mean fluorescence intensity normalized to control levels). (**D**) Western blot analysis for total PKCα (Cell Signaling #2056, polyclonal) and phospho-PKCα (Thr638) expression. Densitometric analysis of (**E**) total and (**F**) phosphorylated PKCα expression from Western blot analyses (n ≥ 4 experiments; error bars indicate SD). (**G**) Real-time PCR analysis of KG1a cells. (**H**) Immunofluorescence imaging of CD82 (Biolegend, ASL-24) and PKCα-488 (primary, abcam, Y124; secondary, Invitrogen, rabbit-488) under resting and 1 hr of PMA treatment with corresponding line scan plots for both channels. All channels were scaled equally across conditions. (**I**) Western blot analysis of total and phosphorylated PKCα expression following PMA stimulation (n ≥ 4 experiments; error bars indicate SD). (**J**) Cells were treated with DMSO, PMA or PMA + MG132 (10 uM) for 4 hrs and total and phospho-PKCα were quantified using Western blot analysis and densitometry. (n ≥ 4 independent experiments; error bars indicate SD; post-hoc unpaired t-test).

To identify how CD82 membrane scaffolding affects PKCα signaling, we generated KG1a AML cell lines stably overexpressing wild type CD82 (CD82OE) or a palmitoylation mutant form of CD82 (Palm-CD82OE) tagged to the mCherry fluorescent protein. In the palmitoylation mutant, five membrane proximal cysteine residues are mutated to serines, preventing CD82 palmitoylation (Fig. 1A)^29^. We also generated CD82 knockdown KG1a cells (CD82KD) cells, where stable expression of CD82-specific shRNAs reduces total CD82 expression by 50% and surface levels by 95% (Fig. 1B,C). To quantify total and surface CD82 expression, we used flow cytometry analysis of permeabilized (Fig. 1B) and non-permeabilized cells, respectively (Fig. 1C). We also measured the surface expression of other tetraspanins in these cell lines, finding similar levels of CD9 in all cell lines (Suppl. Fig. 1A), and decreased levels of CD151 (Suppl. Fig. 1B) in the CD82KD cells compared to controls. Interestingly, we find decreased levels of surface (Suppl. Fig. 1C) and total (Suppl. Fig. 1D) CD81 in CD82KD and CD82OE cells compared to control cells. Two additional myeloid leukemia cell lines (K562 and U937) were engineered to overexpress WT-CD82 or Palm-CD82 (Suppl. Fig. 2A–E,I–M). In K562 cells, CD81 and CD151 expression is similar across the cell lines, while CD9 expression is increased in the CD82OE cells (Suppl. Fig. 2F–H). U937 cells also display increased CD9 expression in the CD82OE cells and an increase in both CD9 and CD81 in the Palm-CD82OE cells, whereas CD151 expression remains unchanged (Suppl. Fig. 2N–P). In combination, these data suggest that changes in CD82 expression can regulate the tetraspanin profile expressed in leukemic cells.

To analyze how CD82 scaffolding regulates the expression and activation of PKCα, we first quantified the expression levels of total and activated PKCα using Western blot analysis. (Data presented utilize KG1a cells with additional cell line analysis quantified in supplemental data). Figures 1D–F demonstrate that the CD82OE cells have a twofold increase in total PKCα expression and a 1.3-fold increase in phosphorylated (active) PKCα expression compared to control cells. In contrast, we find that the Palm-CD82OE cells express approximately 50% less total PKCα and 60% less phospho-PKCα when compared to control cells. Similar changes in PKCα expression and activation were identified in the U937 cells overexpressing CD82 (Suppl. Fig. 2Q–R), whereas the K562 cells only display an increase PKCα activation. Upon CD82KD in KG1a cells, we are unable to detect the expression of PKCα or its active form by Western blot (Fig. 1D–F). RT-PCR analysis of the cell lines measures a transcriptional down regulation of PKCα in CD82KD cells and no change in PKCα transcript between the control and CD82OE cells (Fig. 1G). Together, these data suggest a critical role for CD82 expression and membrane organization in regulating PKCα expression and activation in AML.

Upon full activation, PKCα translocates to the plasma membrane from the cytoplasm, which is essential for PKCα signaling. Using immunofluorescence imaging, we find that under resting conditions PKCα is primarily localized within the cytoplasm, whereas, upon PKCα activation with PMA for 1 hr, PKCα translocates to the plasma membrane (Fig. 1H). We also observe by line scan analysis that the intensity plots for the CD82 and PKCα channels have a similar shape under PMA stimulated conditions, suggesting that PKCα activation stimulates PKCα to move to CD82 membrane regions. These data illustrate that despite the CD82 palmitoylation mutation, PKCα effectively translocates to the plasma membrane upon activation.

Following activation, PKCα can be dephosphorylated and degraded in order to down-regulate PKCα-mediated signaling^30^,^31^,^32^,^33^. Therefore, we assessed whether CD82 scaffolding preserves PKCα protein levels upon activation, thereby providing a sustained signal. Upon PMA stimulation for 1 or 4 hrs, we find that total and phospho-PKCα expression is maintained at a higher proportion in the CD82OE cells when compared to Palm-CD82OE cells (Fig. 1I). Next, we investigated if the reduced PKCα expression upon activation is due to proteasomal degradation. Combining 4 hrs of PMA with the proteasomal inhibitor, MG132, we find that PKCα expression is rescued to basal levels in control and Palm-CD82OE cells (Fig. 1J). Collectively, these data suggest that CD82 scaffolding can stabilize PKCα levels upon activation.

### The CD82 scaffold regulates short-term PKCα membrane association

**Figure 2:**
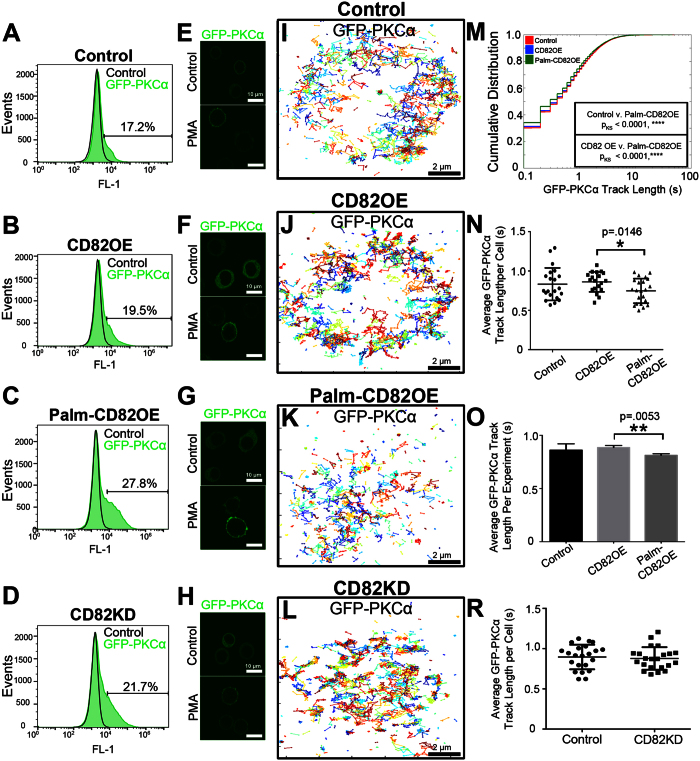
The CD82 scaffold regulates PKCα association with the membrane. (**A**–**D**) Flow cytometry analysis indicates the percentage of GFP-PKCα expression in transiently transfected cells. (**E**–**H**) Epifluorescence imaging of transfected cells showing GFP-PKCα localization +/− PMA. (**I**–**L**) PKCα trajectories from 600 frames of analyses are displayed. (**M**) Cumulative distribution plot of PKCα track length (n ≥ 31227 tracks from n ≥ 19 cells of each kind; the Kolmogorov-Smirnov test was used to compare cumulative distributions). (**N**) Average GFP-PKCα track length per cell and (**O**) per experiment (error bars indicate SD; n ≥ 19 cells, n = 3 experiments; post-hoc unpaired t-test). (**R**) Average track length per cell was quantified in control and CD82KD cells (error bars indicate SD; n = 22 cells).

One mechanism by which the CD82 scaffold could prolong PKCα activation is by stabilizing PKCα membrane recruitment. To visualize the molecular recruitment of PKCα to the plasma membrane upon activation, we performed single particle tracking (SPT) analysis. Using transiently transfected GFP-PKCα cells (Fig. 2A–C) stimulated with PMA (Fig. 2E–G), we analyzed the membrane track length or “dwell time” of GFP-PKCα, which we define as the time between the membrane appearance and disappearance of GFP-PKCα. Figure 2I–K display representative GFP-PKCα trajectories, which were generated by filtering and connecting localizations with the parameters described in the Methods section. A cumulative distribution plot of the GFP-PKCα track lengths indicates that the Palm-CD82OE cells have an increased proportion of short-lived GFP-PKCα tracks compared to control or CD82OE cells, suggesting a shortened PKCα dwell time (Fig. 2M). We also quantified PKCα dwell time based on the average track length per cell analyzed (n ≥ 19 cells) (Fig. 2N) or per independently performed experiment (n = 3 experiments) (Fig. 2O), finding the same trend observed in our cumulative distribution plot. Interestingly, when analyzing GFP-PKCα dwell time in the CD82KD cells (Fig. 2D,H,L), we are unable to detect a change in track length (Fig. 2R), suggesting a potential compensatory scaffold function from other tetraspanins in the CD82KD cells, which may be inhibited by the palmitoylation mutant form of CD82. In combination, these data suggest that CD82 scaffolding has a modest effect on the initial membrane recruitment of PKCα.

### PKCα is recruited and maintained at the CD82 scaffold upon stimulation

**Figure 3:**
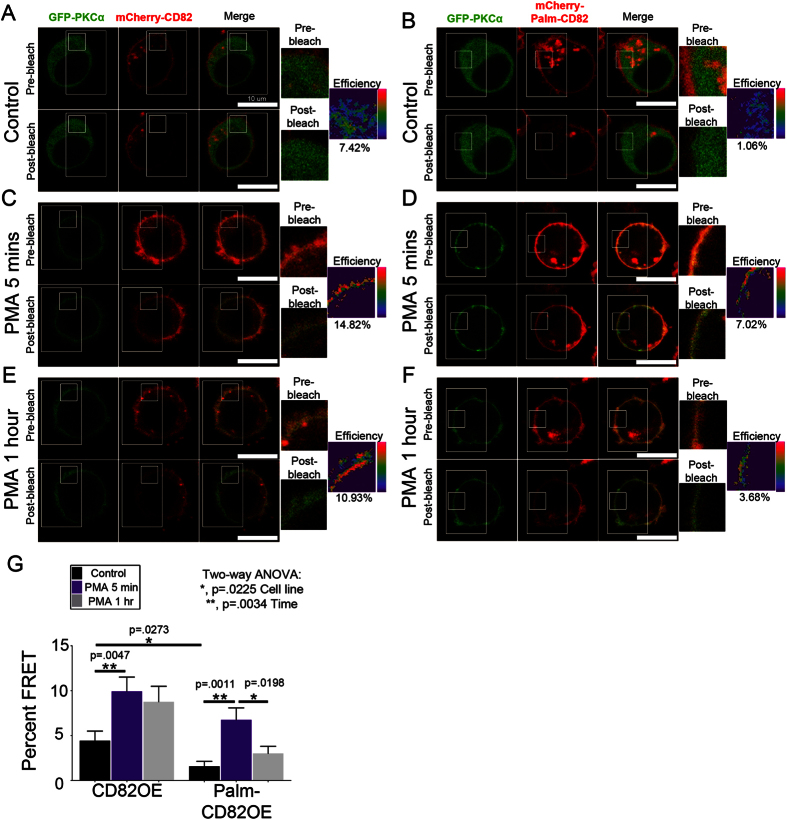
PKCα is stabilized by the CD82 scaffold. CD82OE or Palm-CD82OE KG1a cells were transfected with GFP-PKCα and imaged under (**A**,**B**) resting or upon PMA stimulation for (**C**,**D**) 5 mins or (**E**,**F**) 1 hr. (**G**) Percent FRET efficiencies were calculated in a region of interest per cell. (n = 4 experiments, n ≥ 21 cells per treatment, error bars indicate SEM, post-hoc unpaired t-test).

An extensive series of immunoprecipitation studies demonstrated that upon PMA stimulation, PKCα interacts with CD82^26^, although little is known about the dynamics of this interaction. Our SPT analyses suggest that CD82 palmitoylation may regulate the membrane stabilization of PKCα on a short time scale. However, we are particularly interested in whether CD82 scaffolding can stabilize long-lived PKCα membrane interactions, which could potentiate prolonged signal transduction. Using Förster resonance energy transfer (FRET), we measured the recruitment and retention of PKCα relative to the CD82 scaffold over time. FRET was measured by quantifying fluorescence intensity changes in the donor fluorophore (GFP-PKCα) after the acceptor (mCherry-CD82) was photobleached. CD82OE and Palm-CD82OE cells transiently transfected with GFP-PKCα were imaged under resting conditions and upon PMA stimulation for 5 mins or 1 hr to assess both short and long-term PKCα recruitment, respectively. Under resting conditions, we detect minimal FRET between CD82 and PKCα in both the CD82OE and Palm-CD82OE cells, although the CD82OE cells have higher basal FRET than the Palm-CD82OE cells (Fig. 3A,B,G). Upon PMA stimulation, FRET is significantly increased in the CD82OE and Palm-CD82OE cells compared to resting cells (Fig. 3C,D,G), indicating that PKCα interacts with both the wild type and palmitoylation mutant form of CD82 upon activation. After of 1 hr of stimulation, we find that the increased FRET efficiency is maintained in the CD82OE cells, whereas the FRET is significantly reduced in the Palm-CD82OE cells over the same timeframe (Fig. 3E–G). These data suggest that disruption of the CD82 scaffold, in the case of the palmitoylation mutant, reduces the membrane association of PKCα with CD82. Together, these findings demonstrate that CD82 and PKCα have a prolonged membrane interaction that is hyperstabilized by overexpression of the CD82 scaffold.

### PKCα clustering at the membrane is controlled by the CD82 scaffold

**Figure 4:**
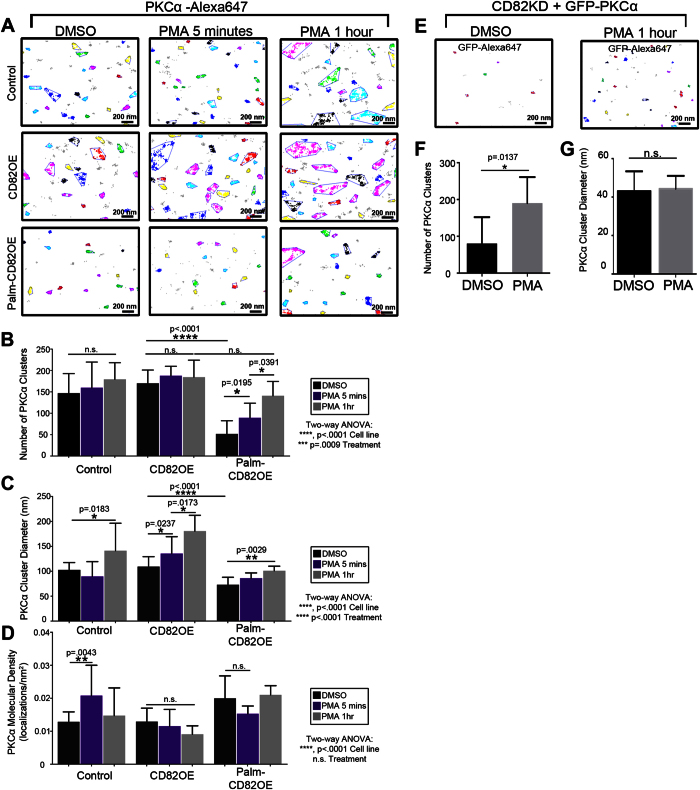
PKCα clustering at the membrane is controlled by the CD82 scaffold. Control, CD82OE and Palm-CD82OE KG1a cells were treated with DMSO, or PMA for 5 mins or 1 hr and imaged for PKCα (abcam, Y124; Invitrogen, rabbit-647) using dSTORM. (**A**) The DBSCAN algorithm was used to examine cluster organization within a subregion of the cells. Clustered localizations are indicated by color, whereas gray localizations did not meet the clustering parameters (ε = 50 nm, n = 30 localizations). The DBSCAN algorithm was used to determine the (**B**) number of PKCα clusters, (**C**) PKCα equivalent cluster diameter, and (**D**) PKCα molecular density (n ≥ 4 cells of each condition, error bars indicate SD, post-hoc unpaired t-test). CD82KD cells were transfected with GFP-PKCα and imaged using dSTORM. (**E**) PKCα clustering was quantified using the DBSCAN clustering algorithm in cells treated with DMSO or PMA (ε = 50 nm, n = 10 localizations). (**F**) The number of clusters (n = 7 cells, error bars indicate SD) and (**G**) the cluster diameters were quantified (n ≥ 561 clusters, error bars indicate SEM, unpaired t-test).

Tetraspanins can regulate the clustering of membrane proteins^34^,^35^,^36^. Interestingly, PKC has also been shown to oligomerize^37^ and aggregate upon activation^38^. Therefore, we next wanted to determine how altered interactions between CD82 and PKCα described in our FRET studies could modulate PKCα clustering. Using the super-resolution imaging (SRI) technique, direct stochastic optical reconstruction microscopy (dSTORM), we resolved the molecular landscape of PKCα in control, CD82OE and Palm-CD82OE cells stimulated with PMA for 5 mins or 1 hr. The organization of signaling proteins into clusters may stabilize signaling by providing steric protection from negative regulators^39^. Therefore, we applied the density-based spatial clustering of applications with noise (DBSCAN) algorithm^40^ to our SRI data to quantify PKCα clustering (Fig. 4A). Under resting conditions, we detect a similar number of PKCα clusters between control and CD82OE cells, whereas the Palm-CD82OE cells display a significantly reduced number of PKCα clusters compared to control and CD82OE cells (Fig. 4B). Next, upon PMA stimulation for 5 min or 1 hr, we again measure no significant change in the number of PKCα clusters in either the control or CD82OE cells. However, in the Palm-CD82OE cells, PMA stimulation results in a significant increase in PKCα cluster number (Fig. 4B). In fact, upon PMA stimulation for 1 hr, the control, CD82OE and Palm-CD82OE cells all exhibit similar numbers of PKCα clusters (Fig. 4B). These data suggest that while Palm-CD82OE cells have reduced PKCα clusters under basal conditions, PMA treatment stimulates a similar number of PKCα clusters in all cells.

It has been previously suggested that the size of signaling molecule clusters is predicted to have a significant impact on signal transduction^39^. Therefore, we next addressed how CD82 scaffolding affects PKCα cluster size. Further analysis of the DBSCAN data indicates that under resting conditions, PKCα cluster diameter is similar between control and CD82OE cells, but is reduced in the Palm-CD82OE cells (Fig. 4C). Upon PMA stimulation for 5 min, only the CD82OE cells exhibit an increase in PKCα cluster area. However, upon PMA stimulation for 1 hr, all the cells increase their PKCα cluster size with the CD82OE promoting even larger “superclusters”^41^.

Additionally, we assessed how CD82 scaffolding modulates PKCα molecular density, or the number of PKCα localizations found per cluster area, since this is another mechanism by which PKCα may be recruited into clusters upon activation. Our data demonstrate that upon PMA stimulation for 5 mins, control cells display increased PKCα molecular density compared to resting conditions (Fig. 4D). Meanwhile, the other cell lines exhibit similar PKCα molecular density upon resting or stimulated conditions. These data suggest that CD82 concentration affects the means by which PKCα is initially recruited to the membrane. More specifically, in the case of the control cells, a lower concentration of CD82 results in PKCα recruitment into densely packed clusters initially (5 m PMA) rather than larger-order superclusters as observed with CD82 overexpression. However, prolonged activation (1 hr PMA) continues to increase the PKCα molecules recruited to the membrane, ultimately leading to increased PKCα cluster sizes (Fig. 4B). These data demonstrate that CD82 concentration and scaffolding properties regulate unique aspects of PKCα membrane clustering.

We also assessed PKCα clustering in the CD82KD cells by transiently transfecting in GFP-PKCα and performing SRI analyses (Fig. 4E). Upon PMA stimulation for 1 hr, we measure an increase in the number of PKCα clusters, consistent with PKCα membrane translocation (Fig. 4F). However, in contrast to the other cell lines, PKCα cluster area remains unchanged in the CD82KD cells following PMA activation (Fig. 4G), suggesting that the CD82 scaffold is necessary to promote or stabilize the larger PKCα clusters measured following PMA stimulation. Combined, these data demonstrate that CD82 scaffolding significantly impacts PKCα cluster size.

### CD82 modulates ERK1/2 activity downstream of PKCα stimulation

**Figure 5:**
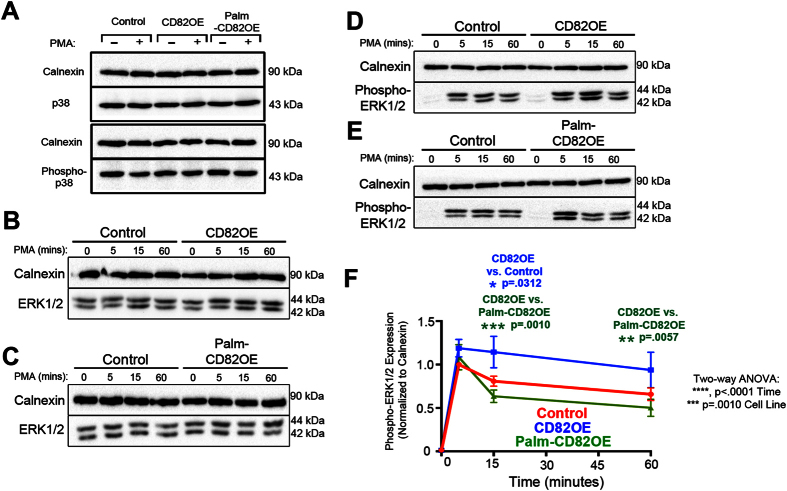
CD82 modulates ERK1/2 activity downstream of PKCα stimulation. (**A**) Control, CD82OE and Palm-CD82OE cells were treated with DMSO or PMA for 1 hr and analyzed by Western blot analysis for total (D13E1) and phospho-p38 (Thr180/Tyr182). Representative Western blot showing control and (**B**) CD82OE cells or (**C**) Palm-CD82OE cells treated with PMA for 0, 5, 15, or 60 mins and analyzed for total ERK1/2 (137F5) expression. Representative Western blot depicting (**D**) control and CD82OE or (**E**) Palm-CD82OE cells treated with PMA for 0, 5, 15, or 60 mins and analyzed for phospho-ERK1/2 (Thr202/Thr204) expression. (**F**) Graphical depiction of phospho-ERK expression over time quantified by Western blot analysis. (n ≥ 4 experiments, error bars depict SEM; post-hoc unpaired t-test).

The ability of PKCα to propagate a signal is dependent upon activation and sufficient membrane recruitment, which allows PKCα to phosphorylate a substrate and elicit a downstream response. Our findings suggest that CD82 stabilizes PKCα at the plasma membrane and promotes larger-scale clustering. We next examined how this stabilization and clustering affects PKCα–mediated signal propagation. One pathway that has been studied extensively with respect to PKCα is the MAPK pathway. Incidentally, it has been shown that MAPK can be constitutively active in leukemias and targeting this activation can help to promote AML blast susceptibility to apoptosis^42^. To determine how CD82 scaffolding affects PKCα-mediated signaling through MAPK, we stimulated cells with PMA and monitored p38 and ERK1/2 activation. Western blot analysis indicates that p38 expression and activation remain unchanged following PMA stimulation in all cell lines (Fig. 5A). Moreover, we find no change in total ERK1/2 expression between the cells (Fig. 5B,C) and detect only minimal phospho-ERK1/2 expression in unstimulated cells (Fig. 5D,E). However, upon PMA stimulation, phospho-ERK1/2 expression varies substantially between the cells. We find that there is increased phospho-ERK1/2 expression in the CD82OE cells compared to control and Palm-CD82OE cells upon 15 mins of PMA stimulation (Fig. 5D–F). Interestingly, the CD82OE cells maintain significantly higher phospho-ERK expression upon 1 hr of PMA stimulation compared to Palm-CD82OE cells (Fig. 5F). Together, these data demonstrate that CD82 scaffolding is critical for regulating the signaling kinetics of ERK1/2 downstream of PKCα activation.

### CD82 regulates AML colony formation in a PKCα-dependent manner

**Figure 6:**
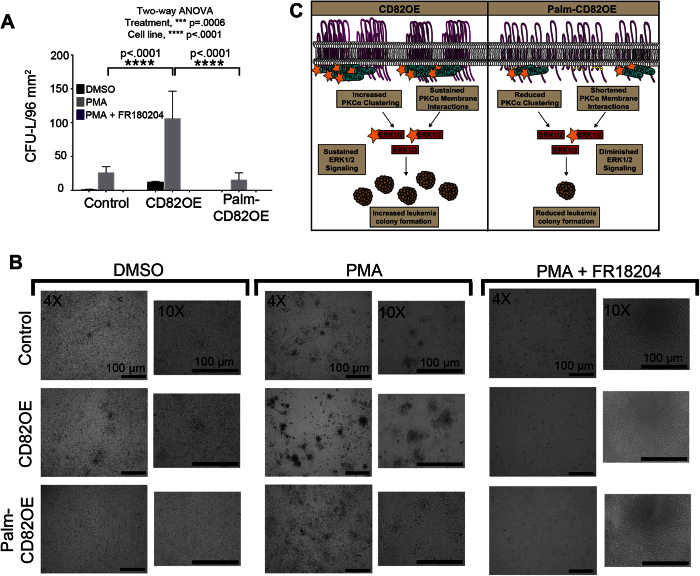
CD82 regulates AML colony formation in a PKCα-dependent manner. (**A**,**B**) Control, CD82OE and Palm-CD82OE cells grown in clonogenic assays in the presence of PMA alone (10 ng/ml), or PMA + FR180204 (100 μM), or equal volumes of DMSO and assessed after 14 days by microscopy for the number of leukemia colony-forming units per 96 mm^2^ (n ≥ 4 experiments, error bars indicate SD). (**C**) Proposed model whereby the scaffolding function of CD82 regulates the membrane clustering and stabilization of PKCα, which controls ERK1/2 signaling and AML colony forming potential.

Finally, we wanted to determine how PKCα activation and ERK signaling affect the leukemia colony forming potential of AML cells. We treated cells with DMSO, PMA, or PMA in combination with the ERK1/2 inhibitor, FR180204. Cells were then plated in MethoCult H4334 media for 14 days, after which, the leukemia colony-forming units (CFU-L) were counted via microscopy. Following PMA treatment, we find that all cells increase CFU-L formation with the CD82OE cells displaying more than four times as many colonies compared to control and Palm-CD82OE cells (Fig. 6A,B). Interestingly, the combined treatment of PMA and ERK1/2 inhibitor significantly reduced colony growth in all cells lines. These data indicate that the CD82- and PMA-mediated increases in CFU-L formation are dependent upon ERK1/2 signaling. Collectively from these data, we suggest the current model (Fig. 6C) where the CD82 scaffold recruits and stabilizes PKCα in membrane clusters, which can sustain ERK1/2 signaling for the development of an aggressive leukemia phenotype.

## Discussion

In this study, we provide new insights into how tetraspanins can serve as membrane scaffolds that control signal transduction in AML. As PKCα is a critical signaling hub for controlling AML cell proliferation and survival^3^, we focused on identifying the properties of tetraspanins that contribute to aberrant PKCα signaling. Numerous studies defined an interaction between PKCα and tetraspanins, but the mechanisms regulating this association and the downstream signaling consequences remain poorly understood. Our study describes a role for CD82 membrane organization in regulating PKCα expression, membrane stabilization and signaling.

Increased phospho-PKCα expression has been correlated with poor survival rates in AML patients^4^ and increased AML cell viability^11^. Consistent with previous findings^43^, data from our study demonstrate that the overexpression of CD82 increases total and phospho-PKCα expression (Fig. 1D–F). Upon mutation of the palmitoylation sites within CD82, we detect decreased total and phospho-PKCα expression (Fig. 1D–F) when compared to control or CD82OE cells, suggesting that the scaffolding properties of CD82 modulate PKCα expression. Previous studies^43^ observed a similar decrease in PKCα expression upon knock down of CD82, further supporting the importance of the CD82 scaffold for maintaining PKCα expression. Similarly, we detect a reduction in PKCα expression upon CD82KD in our study, which we link to reduced PKCα transcript levels. At this point, the mechanism behind this reduction in PKCα mRNA in the CD82KD cells remains undefined. In contrast, we detect similar PKCα transcript levels between the control, CD82OE and Palm-CD82OE cells (Fig. 1G). As palmitoylation has been demonstrated to regulate TEM organization^14^,^44^, we speculate that TEM disruption by CD82 mutation renders PKCα more susceptible for degradation.

Interestingly, another difference identified between the CD82OE and Palm-CD82OE cells is the expression of CD81, which has been shown to interact with PKCα^26^ and is significantly reduced in the CD82OE cells (Suppl. Fig. 1C,D). We speculate that compensatory roles of CD81 and CD82 within TEMs may result in the decreased CD81 expression in the CD82OE cells. Perhaps to maintain the appropriate tetraspanin concentration, scaffold structure, or functional TEM signaling, CD81 is down regulated in response to the overexpression of CD82. However, in the Palm-CD82OE cells, which have disrupted TEMs^36^, CD81 expression may be maintained in an effort to conserve the functionality of these domains. Additionally, since CD81 is a PKC interacting protein, its expression may be maintained in the Palm-CD82OE cells in an attempt to preserve PKCα membrane recruitment and signaling under conditions where PKCα expression is diminished.

A number of previous studies have proposed that tetraspanins serve as protein recruitment platforms. For example, the presence of CD82 was shown to enhance the PKCα phosphorylation of c-Cbl following HB-EGF activation, which led the authors to suggest that CD82 could in fact serve to recruit PKCα^45^. Additionally, a described role for CD151 was to recruit PKCα into proximity with the α6β4 integrin, which significantly impacted tumor initiation and progression^46^. Our SPT data suggest a decrease in PKCα membrane dwell time in the Palm-CD82OE cells (Fig. 2M–O), indicating that disruption of the CD82 scaffold organization may shorten PKCα membrane interactions. Our PKCα tracking experiments used GFP, which has a relatively short fluorescence lifetime. As such, we detect sub-second PKCα track lengths, which may contribute to the modest change seen in PKCα track length in the Palm-CD82OE cells. More pronounced track length differences might be observed with the use of a more stable tracking probe. Additionally, the differential CD81 expression observed in the cell lines may mask a more substantial change in PKCα membrane stabilization as detected with SPT. Despite experimental limitations, our data suggest that CD82 organization alters the interaction of PKCα at the membrane.

Biochemical characterization of tetraspanins suggests that PKCα and PI4K may have distinct tetraspanin recruitment sites, indicating the potential for differential recruitment of signaling enzymes to specific tetraspanins^26^. One possible explanation for how decreased PKCα dwell time could occur is through diminished interactions with the CD82 signaling platform. Our FRET analyses (Fig. 3) indicate that PKCα interacts with both wild type and Palm-CD82, demonstrating that CD82 palmitoylation is not essential for the interaction to occur. However, following 1 hr of PMA stimulation, the FRET efficiency between PKCα and CD82 is sustained, while it is significantly diminished in the Palm-CD82OE cells. These data suggest that CD82 scaffolding contributes to the long-lived protein interactions between PKCα and CD82 at the membrane. Interestingly, PKCα can also be palmitoylated, which was shown to facilitate its membrane recruitment^47^. Therefore, future studies may be directed at understanding how PKCα palmitoylation contributes to the robust membrane interaction between tetraspanins and PKCα.

While tetraspanins have been described to regulate membrane protein clustering^34^,^35^,^36^, this study explores how tetraspanins modulate cytosolic protein clustering. Previous work has demonstrated that the number and size of Ras clusters contributes to its downstream signaling response^48^,^49^. Moreover, increased expression of galectin-1, a Ras membrane scaffold, can enhance Ras-mediated signaling^50^. The current study has uncovered a role for CD82 in regulating PKCα oligomerization, a concept that was hypothesized to have physiological signaling consequences^37^. By taking advantage of the newly developed SRI techniques, we provide visual and quantitative evidence at the nanometer scale for how tetraspanins regulate the spatial arrangement of cytosolic proteins. First, our data suggest that CD82 scaffolding can modulate PKCα cluster number. In the Palm-CD82OE cells, we detect reduced PKCα clusters until PMA stimulation for 1 hr, which results in a similar number of clusters in all cells (Fig. 4B). These data suggest that a limited number of PKCα membrane recruitment sites exist and that PKCα cluster number is dependent upon both the scaffolding of CD82 as well as the concentration of PKCα.

Additionally, our data implicate CD82 expression and scaffolding as regulators of PKCα cluster size. For example, CD82OE cells support PKCα cluster diameters that are approximately 30% and 80% larger than those in control and Palm-CD82OE cells, respectively (Fig. 4C). It is important to take into account the ratio of surface CD82 to PKCα in our cell lines for interpretation of these data. By setting the control cells at a 1/1 ratio of CD82/PKCα, the CD82OE cells have a ratio of 2/2, while the Palm-CD82OE cells have a ratio of 2/0.5. We suggest that the additional CD82 expression in CD82OE cells creates a larger platform to enhance the size of PKCα clusters formed at the membrane. Despite the fact that the Palm-CD82OE cells have the same concentration of CD82, the scaffolding capacity is disrupted and the PKCα concentration is reduced, resulting in smaller PKCα clusters observed. Therefore, it appears that the concentration of CD82 and its scaffold organization work in concert to provide a membrane platform to regulate the recruitment, stability, and cluster size of PKCα.

Aberrant activation of the ERK pathway is implicated in AML progression^51^. Previous studies have shown that inhibiting MAPK signaling in AML can lead to increased apoptosis and reduced proliferation^52^,^53^,^54^,^55^. Additional studies have shown that the treatment of lymphoid cells with CD81 and CD9 antibodies modulate proliferation through alterations in the ERK1/2/MAPK pathway^12^,^56^,^57^. Our data indicate that increased expression of CD82 results in a robust and sustained activation of ERK1/2 upon PMA stimulation that is maintained out to 1 hr (Fig. 5F). However, in the Palm-CD82OE cells, the ERK1/2 activation is abrogated to approximately 50% of the CD82OE response at 1 hr following PMA stimulation. We postulate that the sustained levels of activated PKCα in the CD82OE cells (Fig. 1I) serve to stimulate and maintain the activation of ERK1/2. Conversely, we suggest that the reduced levels of PKCα seen in the Palm-CD82OE cells upon activation (Fig. 1I) leads to a quick turnover in ERK1/2 signaling. It has been hypothesized that membrane clustering of signaling molecules can regulate signal transduction, with smaller, short-lived “nanoclusters” responsible for rapid signaling and larger “microclusters” promoting sustained signal transduction^39^. Our findings are consistent with this notion, demonstrating that increased PKCα “microcluster” formation seen in the CD82OE cells (Fig. 4C) correlates with sustained ERK1/2 signaling. Our findings duggest that CD82 scaffolding primarily affects the long-lived phase of ERK signaling, which further implicates that the CD82-mediated effects on the spatial and temporal dynamics of PKCα can significantly impact the prolonged downstream ERK1/2 effector signaling.

ERK activity has been linked to cell proliferation and leukemia chemoresistance^58^,^59^. Additionally, CD82 expression was shown to be increased in the chemotherapy-resistant CD34^+^/CD38^−^ cells in AML^28^. Our leukemia colony-forming unit assays indicate that CD82OE cells form significantly more AML colonies when compared to control or Palm-CD82OE cells, suggesting that CD82OE cells have a colony forming advantage independent of PKCα stimulation. Interestingly, following PMA treatment, CD82OE cells generate an even greater increase in leukemia CFU formation, indicating that PKCα activation and downstream signaling regulate the aggressiveness of AML. Moreover, we measure a significant reduction in CFU-L formation upon treatment with the ERK1/2 inhibitor FR180205, suggesting that both CD82- and PMA-mediated colony formation are dependent on ERK1/2 signaling. Together, these data suggest that targeting the CD82 scaffold may provide an alternative route towards regulating PKCα and its downstream signaling response in AML. Tetraspanins are already being used in clinical trials for the treatment of chronic lymphocytic leukemia^60^. Therefore, the ability to specifically disrupt the CD82 membrane organization, where aberrant signaling can be initiated and sustained, may represent a novel approach to the treatment of AML.

## Methods

### Cell Culture

The KG1a, K562 and U937 cell lines (American Type Culture Collection) were cultured in RPMI 1640 medium supplemented with 10%FBS, 2 mM l-glutamine, 100 u/ml penicillin, and 100 μg/ml streptomycin. Cells were incubated at 37 °C, 95% humidity, and 5%CO_2_. For stimulation experiments, cells were treated with 10 ng/ml of PMA alone (Sigma), or combined with FR180204 (Tocris) at 100 μM or equivalent volumes of DMSO.

### Plasmids/Cell Line Generation

The mCherry-CD82 and mCherry-Palm-CD82 plasmids were constructed as previously described^36^. Cells were nucleofected with the aforementioned plamids or the mCherry-C1 plasmid (Invitrogen) and then sorted for mCherry expressing cells using fluorescence activated cell sorting at the Flow Cytometry Facility, UNMHSC to generate a stable pool and kept under selection using 500 μg/ml of G418. Stable CD82 knockdown was established using KG1a cells transfected with the CD82 shRNA plasmid (Santa Cruz Biotechnology, sc-35734-SH); cells were put under puromycin selection for 4 weeks and sorted for negative CD82 surface expression. The GFP-PKCα plasmid, cloned in the pEGFP-N3 vector, was generously provided by Dr. Yousuf Hannun from Stony Brook University, Stony Brook, NY. Cells were transiently nucleofected with GFP-PKCα according to the manufacturer’s protocol (Amaxa, Lonza Group).

### Western Blotting

Western blots were performed as previously described^36^. Antibodies used for Western blotting were purchased from Cell Signaling Technology as follows: calnexin (C5C9), PKCα (#2056, polyclonal), phospho-PKCα (Thr638), ERK1/ERK2 (137F5), phospho-ERK1/ERK2 (Thr202/Thr204), p38 (D13E1), phospho-p38 (Thr180/Tyr182), or β-Actin (Sigma, AC-74); all antibodies were used at a 1:1000 dilution. Horseradish peroxidase conjugate enzymes were stimulated with SuperSignal West Pico Chemiluminescent Substrate or Femto Maximum Sensitivity Substrate (Life Technologies). Blots were imaged using the ChemiDoc XRS Imager (Bio-Rad) and analyzed using ImageJ (National Institutes of Health) densitometry software.

### Flow Cytometry

For surface expression, cells were labeled with antibody or the corresponding isotype control in 1%BSA/PBS for 30 mins on ice. For total expression, cells were fixed with 4% paraformaldehyde and blocked with 1%BSA/PBS/0.2%Tween for 1 hr before labeling. Cells were washed 3 times and analyzed using an Acuri C6 flow cytometer; histograms were generated using FlowJo software. Mean fluorescence values were normalized to the “control” cell line level. Antibodies used were CD82-647 (Biolegend, ASL-24), CD81-FITC (Biolegend, 5A6), CD151-PE (BD Biosciences, 14A2.H1), and CD9-647 (Bio-Rad, MM2/57).

### Real-time PCR

The TRIzol Reagent protocol was used to isolate total RNA; cDNA was synthesized using qScript cDNA SuperMix protocol. Fast SYBR Green Master Mix was used for PCR reaction. The following primers were used for amplification: PKCα forward: 5′ ATC CGC AGTGGA ATG AGT CCT TTA CAT 3′, PKCα reverse: 5′ TTG GAA GGT TGT TTC CTG TCT TCA GAG 3′, GAPDH forward: 5′-GTCGGTGTCAACGGATTT-3′, human GAPDH reverse: 5′-ACTCCACGACGTACTGAGC-3′. The PCR plate was read using the 7500 Fast Real-Time PCR System (Applied Biosystems). The Ct value from the sample was normalized to the expression of *GAPDH*. Expression values were averaged from three independent experiments and expression level changes were calculated using the 2^−ΔΔCT^ method.

### Immunofluoresence

Cells were fixed with 4% paraformaldehyde and then blocked/permeabilized with 1%BSA/PBS/0.2%Tween. Cells were then incubated with primary antibodies (CD82-Alexa647, 1:125, Biolegend ASL-24; PKCα, 1:200, abcam, Y124). Cells were then labeled with a rabbit-Alexa488 secondary antibody (1:200, Invitrogen). Cells were imaged by laser scanning confocal microscopy with a Zeiss Axiovert 100 M inverted microscope (LSM 510) system (Carl Zeiss, Jena, Germany) using an excitation wavelength of 488 or 633 nm and a 63X/1.2 numerical aperture oil immersion objective. Image analysis was performed using the Zeiss LSM 510 software.

### Super-Resolution Microscopy

Cells were plated on chamber slide wells that were treated with fibronectin (25 μg/ml, Millipore). Cells were fixed with 4% paraformaldehyde and blocked/permeabilized (1%BSA/PBS/0.2%Tween). Cells were labeled with an anti-PKCα antibody (1:200, abcam, Y124), washed, and incubated with a goat anti-rabbit AlexaFluor647 secondary antibody (1:200; Invitrogen). Cells transfected with GFP-PKCα were labeled with an anti-GFP Alexa647 antibody (Biolegend, FM264G). Cells were washed and post-label fixed with 4% paraformaldehyde. Cells were washed and imaged in dSTORM imaging buffer consisting of 50 mM Tris, 10 mM NaCl, 10% w/v glucose, 168.8 u/ml glucose oxidase (Sigma #G2133), 1404.0 U/ml catalase (Sigma #C9332), and 50 mM MEA, pH8.5. Red reference beads were used to stabilize the sample during imaging; drift corrections were performed using MCL NanoDrive stage controller (Mad City Labs, Nano-CLP100). The sample was imaged for 10,000 frames using a custom TIRF microscope system as described previously^61^ that uses an inverted microscope (IX71, Olympus America Inc.). A 637 nm laser (HL63133DG, Thorlabs) is coupled along with a 405 nm laser (Crystal laser), into two mode fibers and focused onto the objective lens with a 1.45 NA (UAPON 150XTIRF, Olympus America, Inc.) for data acquisition. For imaging, emission light was filtered using bandpass filter (FF01-692/40-25, Semrock) and data was collected on an electron-multiplying charge-coupled device (EMCCD) Camera (iXon 897; Andor Technologies, South Windsor, CT). Pixel size was 106.7 nm. Images were acquired at ~20 ms (50 frames/second) for a 256 × 256 pixel region. All of the instrumentation is controlled by custom-written software in Matlab (MathWorks Inc.). For one color imaging, the 637 nm and 405 nm lasers were used concurrently. The 561 nm laser was used for bead stabilization.

Data collected was then analyzed using a method previously described, where the pixel values are converted to photon counts and a 2D localization algorithm is used to determine the x and y positions of emitters, total photon counts, and the background photon counts^62^. The localized emitters were then put through a series of thresholds of various fitting parameters. The fitting parameters used are maximum background photons = 80 and minimum photons per frame per emitter = 500.

The SuperCluster Matlab software (http://stmc.health.unm.edu/tools-and-data/index.html) was used for SRI cluster analysis using the DBSCAN module; a 6 μm × 6 μm region of a cell was analyzed with DBSCAN which outputs the number of clusters and their corresponding areas. Clusters in Fig. 4A–D were determined as having at least 30 localizations within a 50 nm search radius, while clusters in Fig. 4E–G required 10 localizations. Areas were calculated using a convex hull around all points identified as a cluster. Equivalent diameter of a cluster was calculated as area = pi*R ^ 2. Molecular density is calculated as the number of localizations in a cluster divided by the cluster area.

### Förster resonance energy transfer (FRET)

Stable KG1a cells were transfected with GFP-PKCα and plated on 25 μg/μl of fibronectin overnight. Cells were imaged using the Leica SP8 System using a 63X water objective equipped with an objective heater which maintained samples as 34 °C throughout imaging. The excitation light source was a white-light laser system set at 488 nm (GFP) and 561 nm (mCherry). Fluorescence from the 488 nm channel was collected using a HyD1 detector and fluorescence from the 561 nm channel was collected using the HyD SMD2 in standard mode. Photobleaching was performed at 100% 561 nm laser power for 5 frames. GFP and mCherry levels in cells outside of the field of bleaching demonstrate that inherent photobleaching did not play a significant role in reducing GFP or mCherry fluorescence over the course of imaging (Supplemental Fig. 4). FRET efficiencies were calculated using the formula: Efficiency = (Donor_post-bleach_ − Donor_pre-bleach_)/Donor_post-bleach_ where D is the fluorescence intensity in a plasma membrane region of interest of fixed shape and size (3 × 7 ellipse). Analysis was performed using the Leica Application Suite AF Lite software.

### Single Particle Tracking (SPT)

SPT was performed using the TIRF microscope optical setup as described in the “Super-resolution Microscopy” section. A 488 nm laser (Cyan Scientific; Spectra-Physics) was used for GFP excitation. The sample emission light was detected using an EMCCD camera (iXon 897; Andor Technologies). 500 frames per cell were acquired at 20 frames/sec. An objective heater maintained samples as 34 °C throughout imaging.

SPT data processing was performed as described previously^63^. The algorithm first finds box centers from raw data, and then fits these centers to determine the location of single particles. The localizations are then filtered and trajectories are built by connecting localizations. The minimum number of photons to threshold a box was 1.5 photons. Once boxes were determined, the box region size used to determine the localization of single molecules was 7 pixels. In order to filter localizations, the minimum number of photons to consider a localization was 20 photons, while the minimum distance between localized fits was 3 pixels. The maximum number of pixels to search for connections was 8 pixels in x or y. The maximum number of frame gaps to search for connections was 5 frames. The minimum track length to consider valid before gap closing assignments was 2 frames.

### Leukemia Colony-Forming Unit Assay

100,000 KG1a cells were treated with PMA (10 ng/ml) alone, PMA + FR180204 (100 μM) or equivalent volumes of DMSO. Cells were plated in MethoCult H4434 Classic Medium and allowed to grow for 14 days and then leukemia colony forming units (>30 cells) were counted.

### Statistics

Statistical analyses were performed using GraphPad Prism 6 software. For multiple comparisons, one or two-way ANOVA was performed, followed by a Bonferroni multiple comparison analysis Post-hoc unpaired t-tests were performed as referenced, using Welch’s correction if variances were unequal. Alpha = .05 in all analyses. The Kolmogorov-Smirnov test was used to compare cumulative distributions. (*<0.05, **<0.01, ***<0.001, ****<0.0001).

## Additional Information

**How to cite this article**: Termini, C. M. *et al*. Tetraspanin CD82 Regulates the Spatiotemporal Dynamics of PKCα in Acute Myeloid Leukemia. *Sci. Rep.* **6**, 29859; doi: 10.1038/srep29859 (2016).

## Supplementary Material

Supplementary Information
