# Supplementary material for: Tetraspanin CD82 Regulates the Spatiotemporal Dynamics of PKCα in Acute Myeloid Leukemia
Source: Sci Rep. 2016 Jul 15;6:29859. doi: 10.1038/srep29859 (PMC4945921; doi:10.1038/srep29859)
Supplement: Supplementary Information [file srep29859-s1.pdf]

Supplementary information for: “Tetraspanin CD82 Regulates the Spatiotemporal Dynamics of PKCα in Acute Myeloid Leukemia”

Christina M. Termini[1], Keith A. Lidke[2], Jennifer M. Gillette[1*]

1. Department of Pathology, University of New Mexico Health Sciences Center
2. Department of Physics and Astronomy, University of New Mexico,

*Corresponding author, jgillette@salud.unm.edu

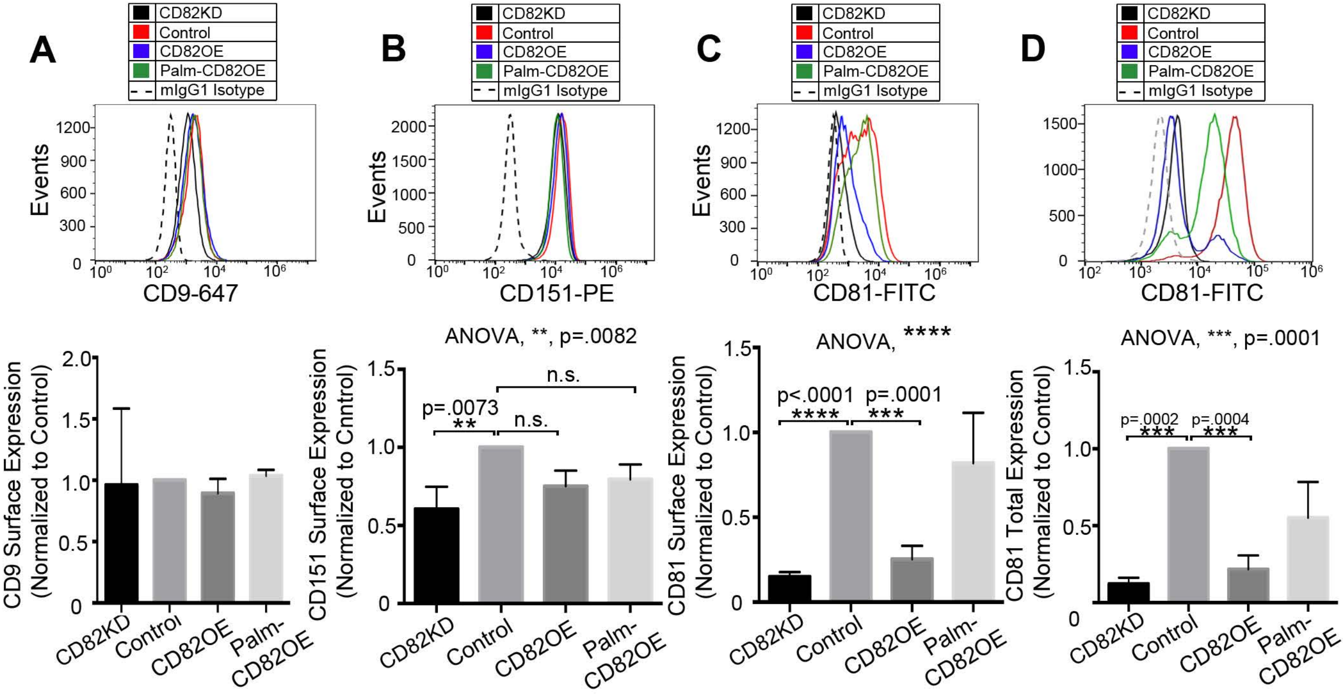

Supplemental Figure 1. CD82 regulates tetraspanin expression levels. Surface expression of tetraspanins assessed in stable KG1a cells using flow cytometry with antibodies specific to A) CD9 (AbdSerotec, MM2/57), B) CD151 (BD Biosciences, 14A2.H1), and C) CD81 (Biolegend, 5A6). D) Permeabilized cells were used to assess total CD81 expression. Quantification of normalized mean fluorescence intensity is depicted below histograms (n≥3 independent experiments, error bars denote SD).

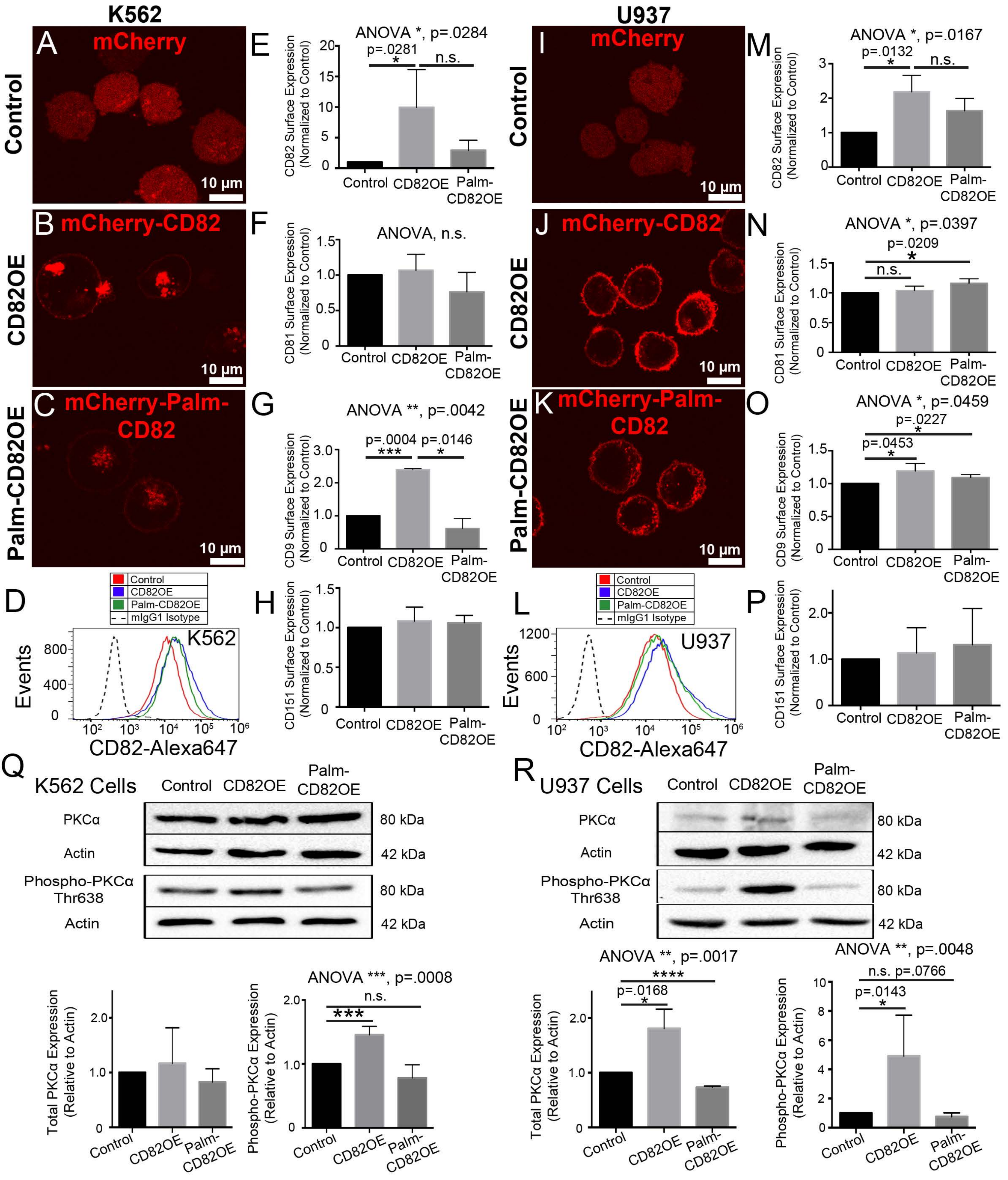

Supplemental Figure 2. CD82 regulates PKCα expression and activation in other AML cell lines. A-C) The mCherry, mCherry-CD82 or mCherry-Palm-CD82 constructs were stably expressed in K562 cells. K562 cells were analyzed for surface expression of D-E) CD82 (ASL-24), F) CD81 (5A6), G) CD9 (MM2/57) and H) CD151 (14A2.H1) (n≥3 experiments, error bars denote SD). I-K) The mCherry, mCherry-CD82 or mCherry-Palm-CD82 constructs were stably expressed in U937 cells. U937 cells were analyzed for surface expression of L-M) CD82 (ASL-24), N) CD81 (5A6), O) CD9 (MM2/57) and P) CD151 (14A2.H1) (n≥3 experiments, error bars denote SD). Q) Western blot analysis of stable K562 cells and densitometry was performed to quantify total and phospho-PKCα expression levels (n≥3 experiments, error bars denote SD). R) Western blot analysis of stable U937 cells and densitometry was performed to quantify total and phospho-PKCα expression levels (n≥3 experiments, error bars denote SD).
